# Mammary fibroblasts reduce apoptosis and speed estrogen-induced hyperplasia in an organotypic MCF7-derived duct model

**DOI:** 10.1038/s41598-018-25461-1

**Published:** 2018-05-08

**Authors:** Molly M. Morgan, Megan K. Livingston, Jay W. Warrick, Eli M. Stanek, Elaine T. Alarid, David J. Beebe, Brian P. Johnson

**Affiliations:** 1 Department of Biomedical Engineering, University of Wisconsin Madison, Madison, Wisconsin USA; 2 Department of Chemistry, University of Wisconsin Madison, Madison, Wisconsin USA; 3 Department of Oncology, University of Wisconsin Madison, Madison, Wisconsin USA

## Abstract

The estrogen receptor (ER) regulates the survival and growth of breast cancer cells, but it is less clear how components of the tissue microenvironment affect ER-mediated responses. We set out to test how human mammary fibroblasts (HMFs) modulate ER signaling and downstream cellular responses. We exposed an organotypic mammary model consisting of a collagen-embedded duct structure lined with MCF7 cells to 17-β estradiol (E2), with and without HMFs in the surrounding matrix. MCF7 cells grown as ductal structures were polarized and proliferated at rates comparable to *in vivo* breast tissue. In both culture platforms, exposure to E2 increased ER transactivation, increased proliferation, and induced ductal hyperplasia. When the surrounding matrix contained HMFs, the onset and severity of E2-induced ductal hyperplasia was increased due to decreased apoptosis. The reduced apoptosis may be due to fibroblasts modulating ER signaling in MCF7 cells, as suggested by the increased ER transactivation and reduced ER protein in MCF7 cells grown in co-culture. These findings demonstrate the utility of organotypic platforms when studying stromal:epithelial interactions, and add to existing literature that implicate the mammary microenvironment in ER + breast cancer progression.

## Introduction

Breast cancer is the most common non-cutaneous cancer in the United States, responsible for approximately 15% of all cancer cases and 7% of all cancer related deaths^1^. Estrogen receptor (ER) signaling regulates the survival and growth of mammary epithelial cells^2^ and is altered in premalignant breast lesions such as atypical ductal hyperplasia (ADH) and ductal carcinoma *in situ* (DCIS), as well as in invasive ductal cell carcinoma (IDC)^3,4^. Consequently, studying ER signaling is a major focus of breast cancer research; ER is a common target for breast cancer therapies^2^, and exposure to ER agonists has been found to increase breast cancer risk^5^.

Classically, *in vivo* models have been used to characterize the effects of ER-targeted cancer therapeutics and toxicants, but are expensive, time consuming, ethically challenging and often lack human relevance^6^. In addition, the complexity of *in vivo* models poses challenges when trying to decipher the molecular and cellular effects of chemicals, as specific interactions are difficult to isolate. Breast cancer models constructed *in vitro* are appealing tools for chemical testing because they are dramatically less resource intensive, more biologically tractable, and could be more relevant to the human condition than *in vivo* animal models. Unfortunately*, in vitro* models often fail to predict *in vivo* response^7,8^, a problem that some have attributed to the oversimplified culture conditions of *in vitro* models^9,10^. Overall, our understanding of how therapeutics and toxicants affect ER + breast cancer is hindered by the lack of available models that are both predictive and resource efficient.

Researchers have speculated that a more physiologically relevant *in vitro* culture environment would push cells to behave more similarly to cells grown *in vivo*, which would increase the predictability of *in vitro* models^10^. The traditional cell culture model evaluates cells grown on a flat plastic surface and lacks components of the mammary microenvironment such as extracellular matrix proteins, tissue structure, or cellular communication; the absence of these components may contribute to the low predictability of *in vitro* models as they could influence ER-driven responses. Mounting studies have found that tissue structure^11–13^, extracellular matrix proteins (ECM)^14,15^, and supportive cell types^16,17^ regulate the behavior of cancer cells, such as invasion, cytokine secretion, and gene expression. There is also evidence that the breast microenvironment regulates ER signaling and function of breast epithelial cells^12,18^. Of particular importance, there is some evidence that mammary fibroblasts regulate ER dynamics in breast cancer cells. For example, mouse mammary fibroblasts have been found to regulate how mouse epithelial cells respond to hormones^19^. While informative, these studies must be cautiously evaluated due to species differences; murine mammary fibroblasts express ERα, where human mammary fibroblasts do not^20,21^. *In vitro* studies using human breast cancer cells have also reported that fibroblasts regulate ER dynamics, where fibroblasts were found to regulate ERα expression and tamoxifen resistance^22,23^. While these studies made important advances, they were confined to two-dimensional (2D) platforms that did not incorporate tissue structure or ECM proteins. Accordingly, pathological endpoints such as ductal hyperplasia, which is a key step in premalignant breast cancer progression, could not be evaluated in these models. In summary, there is a need for physiologically relevant *in vitro* breast cancer platforms that can be used to investigate how components of the mammary microenvironment (such as fibroblasts) affect ER signaling in early stage breast cancer.

Here, we utilized an organotypic mammary model that incorporates a MCF7 cell-derived duct surrounded by a collagen matrix to test the hypothesis that incorporating fibroblasts into the matrix surrounding the duct would lead to differences in estrogen induced cellular responses. We found that fibroblasts modulated epithelial ER protein levels, resulting in increased ER transactivation. Additionally, the incorporation of fibroblasts increased the severity and sped the onset of estrogen-induced hyperplasia. We determined the augmented hyperplasia was due to reduced apoptosis in the co-culture model. Overall, this work presents a structurally relevant platform to evaluate the impact of the mammary microenvironment on ER signaling and suggests that fibroblasts can regulate ER protein as well as apoptosis in breast cancer cells.

## Results

### MCF7s grown in a mammary duct structure form confluent polarized epithelial layers

**Figure 1:**
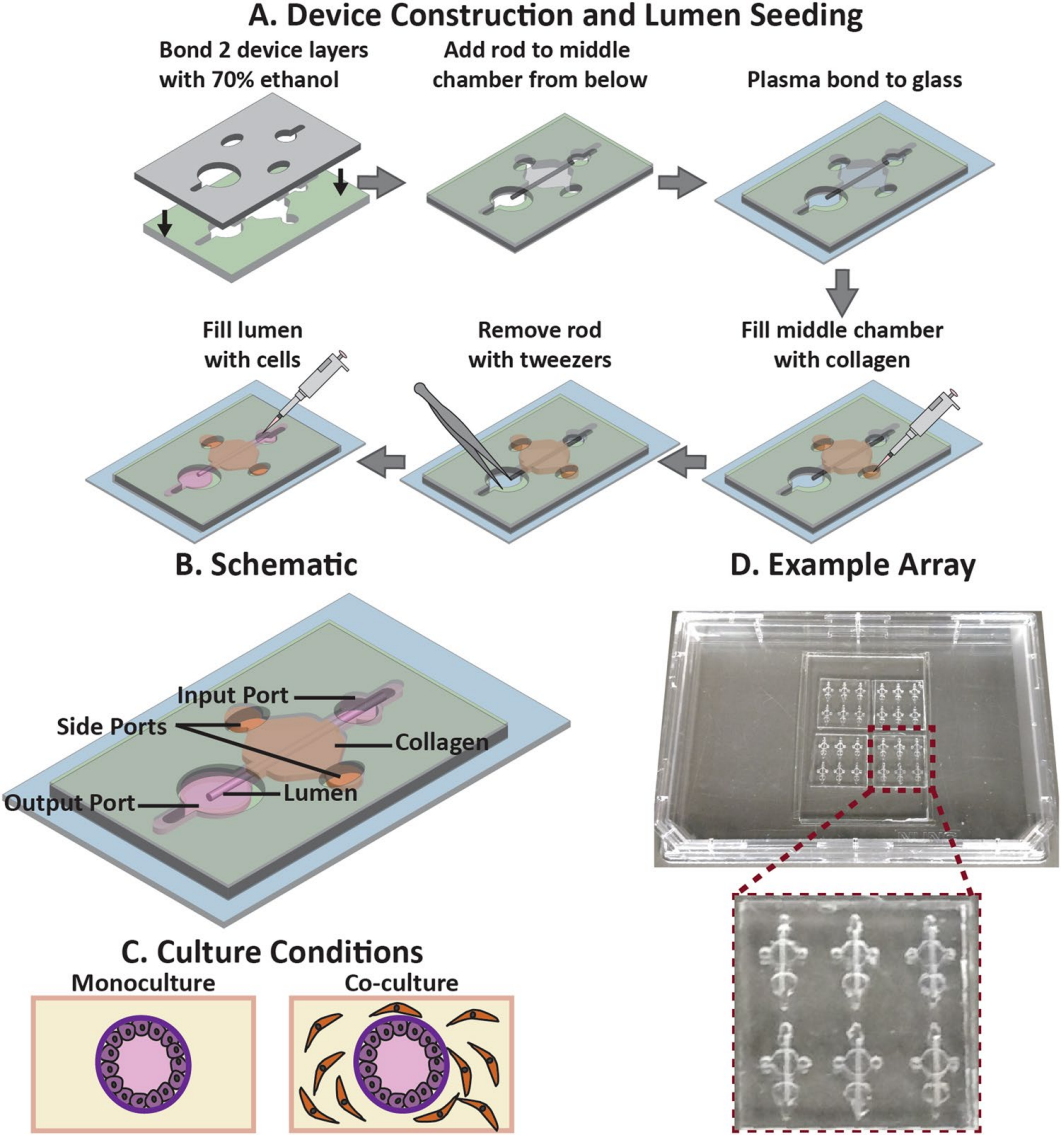
Device illustration and culture conditions. (**A**) The LumeNEXT method^49^ was used to create the two-layered microfluidic devices. (**A**) depicts device construction and use, and (**B**) shows a detailed device schematic. (**C**) The platform allows the investigation of stromal cells on duct function and estrogenic response, as the duct can be cultured in a collagen matrix, as well as cultured in a collagen matrix that has stromal cells embedded throughout. An example of a device array is shown in (**D**), where 24 devices are plasma bonded to a cover slip attached to an omni tray.

Due to previous studies that report the importance of tissue structure on cell behavior^24,25^, the LumeNEXT method (device construction, use and schematic shown in Fig. 1A,B and described in methods) was used to create a lumen structure within a collagen matrix for the base model. To model an early stage breast cancer, the lumen was lined with cancerous mammary epithelial cells and was surrounded by a collagen matrix with or without embedded normal human mammary fibroblasts (HMFs) (shown in Fig. 1C, device example shown in Fig. 1D). Primary mammary epithelial cells rapidly lose ER when grown *in vitro* and consequently are challenging to use for ER-related studies^26^. Therefore, we lined the ductal structures with MCF7s, an immortalized human mammary epithelial cancerous cell line, due to their high expression of ER, their ability to recapitulate hormonal responses similar to ER + breast tumors *in vivo*, and the wealth of literature on MCF7s that can be used to interpret our results^27^.

**Figure 2:**
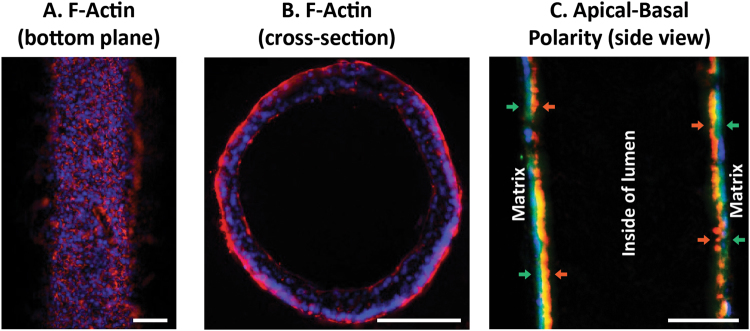
MCF7s form confluent polarized ductal structures. The (**A**) bottom plane and (**B**) cross-section of MCF7-derived lumens stained with F-Actin (red) and nuclei (blue) were imaged. (**C**) To evaluate apical-basal polarity, samples were stained for nuclei (blue), laminin-5 (green; basal), and golgi (red; apical). Scale bars represent 100 μm.

We first characterized the model in the absence of embedded HMFs. To characterize the morphology of the biomimetic duct, MCF7s were grown as ductal structures for 48 hours in normal growth media then were fixed and stained for nuclei and F-Actin. Fluorescent microscopy was used to image the bottom plane of the lumen, which revealed that MCF7s formed confluent epithelial layers within the lumen (Fig. 2A). To investigate cellular pathology, the ducts were embedded in agarose and cross-sectioned which revealed that the cells grow around the periphery of the lumen (Fig. 2B). To evaluate apical-basal polarity, samples were stained for basal marker laminin-5 and apical marker GM130. Laminin-5 was localized to the basal side of the lumen, where GM130 faced the inside of the lumen (Fig. 2C).

Before integration of fibroblasts into the matrix surrounding the mammary duct, the ER status of the HMFs was evaluated. qRT-PCR was performed to evaluate the expression of ERα- and ERβ- driven genes after exposure to 10 nM E2 for 24 hours (Supplemental Fig. 1A). E2 exposure did not induce expression of ER-driven genes in the HMFs. *ESR2* mRNA was also evaluated after treatment with a vehicle control or 10 nM E2, but *ESR2* mRNA was not detected in HMFs of either condition [data not shown]. Immunocytochemistry was used to stain for ERα. In contrast to MCF7s, HMFs did not stain positively for ERα (Supplemental Fig. 1B). We were unable to evaluate ERβ protein because we do not know of a reliable ERβ antibody, which is a known challenge in the field (Nelson *et al*., 2017).

### Fibroblasts reduce ER protein and increase ER transactivation in MCF7s

**Figure 3:**
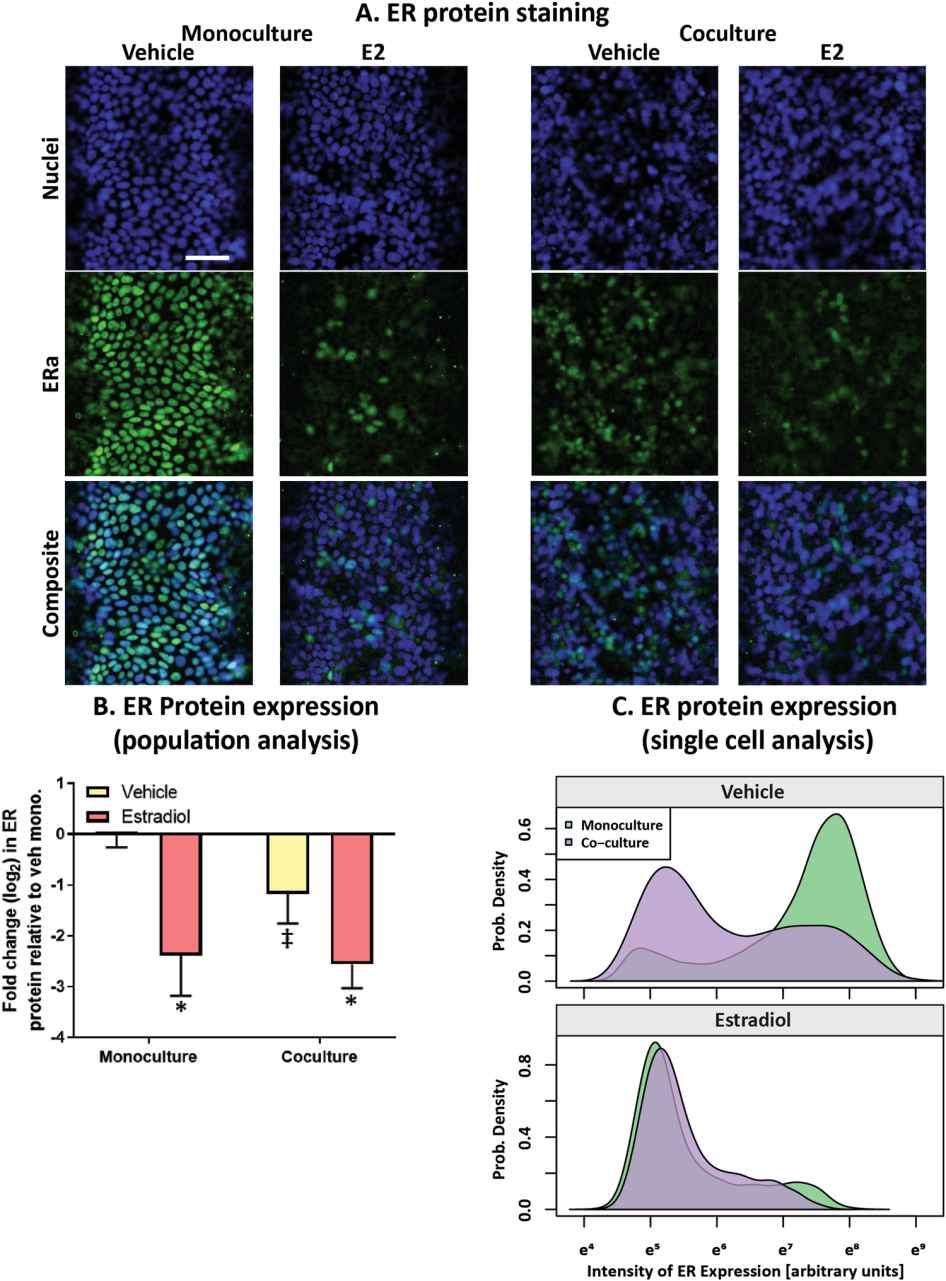
MCF7-derived ducts grown in a mixed collagen:fibroblast matrix express lower levels of ER protein. MCF7-derived ducts were cultured alone or in co-culture with HMFs, treated with a vehicle control or 100 nM E2 for 24 hours. (**A**) Cultures were fixed and stained to visualize nuclei and ERα protein. Scale bar represents 100 μm. To quantify ER protein, the Hoescht stain was used to generate nuclear masks then ER expression was quantified within each nuclear mask. (**B**) Mean ER expression per condition was graphed to visualize ER protein changes within the entire population. A two-way ANOVA was run to evaluate interactions between conditions, followed by a Tukey’s multiple comparison test to identify significant differences. (**C**) A density plot was created to show the frequency of individual cells (x-axis) that expressed various levels of ER protein (y-axis). *= vs. respective vehicle (p < 0.05) and ‡= vs. respective monoculture (p < 0.05). Data is representative of three independent experiments.

As previous studies using 2D culture platforms found changes in ER protein when breast cancer cells were cultured near stromal cells^22,23^, we first evaluated the impact of HMFs on ER protein of the MCF7-derived ducts. Immunocytochemistry was used to evaluate ERα protein after the MCF7-derived duct model was cultured alone or when fibroblasts were cultured in the surrounding matrix, after exposure to a vehicle control or to a saturating concentration (100 nM) of 17β-estradiol (E2) for 24 hours (Fig. 3A). Quantification of ER expression in the bulk cell population (Fig. 3B) as well as in individual MCF7s (Fig. 3C) revealed that vehicle-treated MCF7-derived ducts had decreased ER protein when grown in a mixed fibroblast:collagen matrix, compared to when grown in a collagen only matrix. In both culture conditions, E2 significantly reduced ER protein, which is consistent with previous studies that found ER downregulates its own expression upon activation^28^. Additionally, the estrogen-treated monocultures showed similar levels of ER protein as the estrogen-treated co-cultures. We suspect that the estrogen-treated cultures displayed similar ER protein expression because ER protein is at maximal downregulation in both systems, as the cultures were exposed to a saturating concentration of E2.

**Figure 4:**
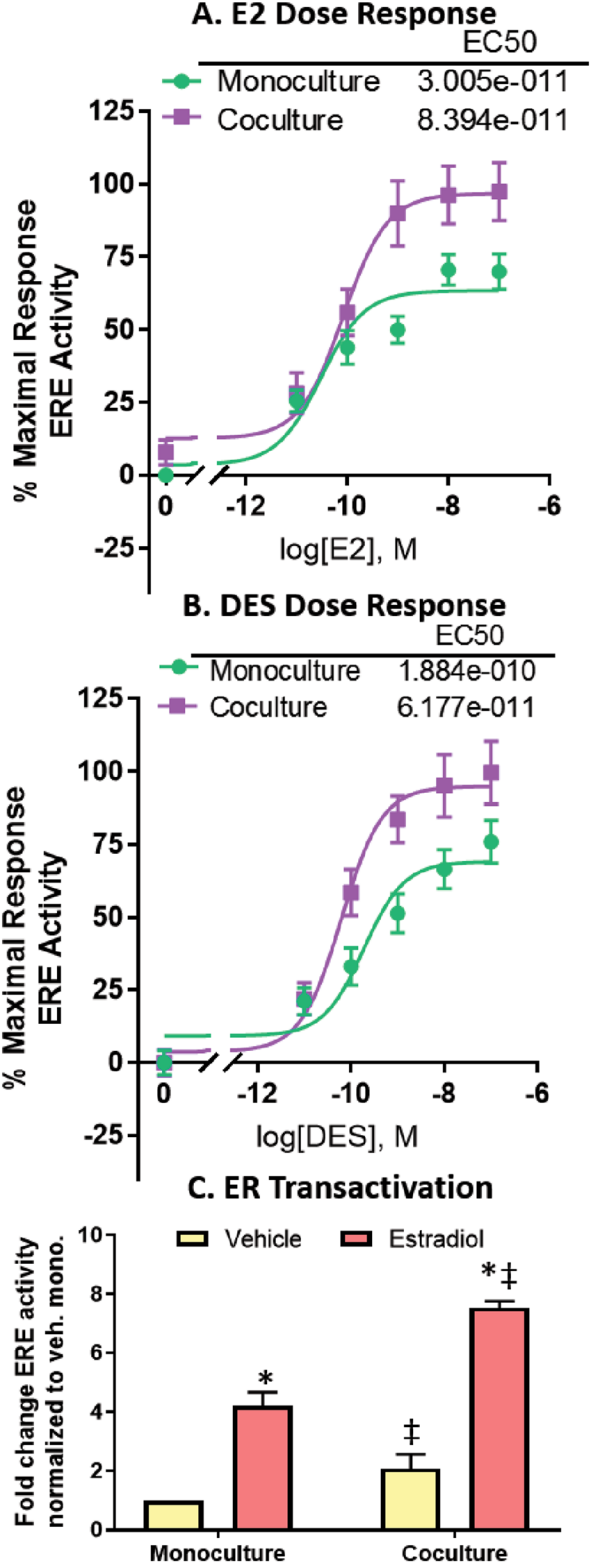
MCF7-derived ducts respond to ER ligands in a dose-dependent manner, and fibroblasts regulate ER transactivation. ER transactivation was evaluated after cultures were exposed to 5 doses of (**A**) E2 and (**B**) DES for 24 hours. EC50s were generated using nonlinear regression. (**C**) ER transactivation in monoculture and co-culture with fibroblasts was evaluated after 48 hours of exposure to a vehicle or 100 nM E2. Luminescent signal was normalized to the total number of nuclei to account for differences in cell number. A two-way ANOVA was run to evaluate interactions between conditions, followed by a Tukey’s multiple comparisons test to identify significant differences. *= vs. respective vehicle (p < 0.05) and ‡= vs. respective monoculture (p < 0.05). Graphs represent data from three independent experiments.

To determine if the differences in ER protein correlated with changes in ER function, MCF7-derived ducts were exposed to five doses of E2 when cultured alone and in co-culture then evaluated for ER transactivation after 24 hours. Upon activation, ER dimerizes and binds to regulatory regions of ER-driven genes called estrogen response elements (EREs), initiating gene transcription. The MCF7s used in the model stably express an ERE-luciferase reporter plasmid; therefore, ER transactivation can be measured by luminescent signal, as luminescence is linear to ERE activity^29^. Both the monoculture and co-culture model responded to E2 in a dose-dependent manner, however, maximal response was slightly increased in the presence of fibroblasts (Fig. 4A). Next, the model was exposed to xenoestrogen diethylstilbestrol (DES) which revealed a similar trend (Fig. 4B). To further investigate the finding that maximal response was increased in co-culture, MCF7-derived ducts were grown in monoculture and co-culture in the presence of a vehicle control or 100 nM E2 for 48 hours. Similar to at the 24-hour time point, the luminescent signal was higher in the co-cultures. Vehicle-treated MCF7-derived ducts co-cultured with surrounding fibroblasts had 4-fold higher ERE activity compared to when cultured alone. There was significantly higher ER transactivation in the E2-treated co-culture compared to the E2-treated monoculture (Fig. 4C).

### MCF7s exhibit increased cell density, reduced apoptosis, and similar levels of E2-induced proliferation in the presence of fibroblasts

**Figure 5:**
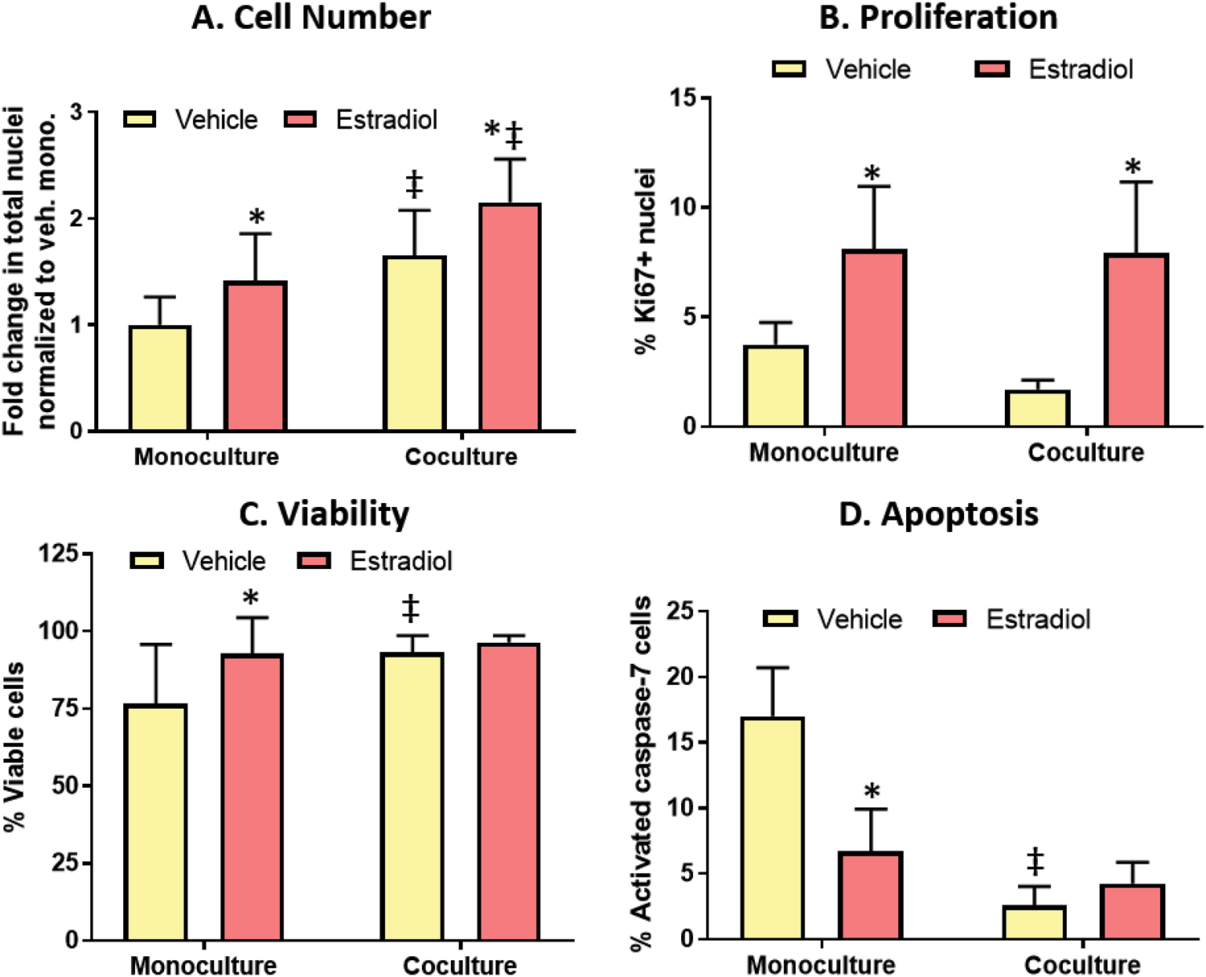
Fibroblasts increase cell number by increasing viability and reducing apoptosis. Cultures were dosed with a vehicle control or 100 nM E2 for 72 hours. (**A**) Total number of nuclei was measured to evaluate cell number. (**B**) Proliferation was measured by the percentage of cells positive for proliferation marker Ki67. (**C**) A live/dead assay was used to evaluate cell viability, where the cells were stained with calcein, Hoescht, and ethidium homodimer. (**D**) Cell apoptosis was measured by quantifying the number of nuclei positive for activated caspase-7, which is a key protein in the apoptosis cascade. A two-way ANOVA was run to evaluate interactions between conditions, followed by a Tukey’s multiple comparisons test to identify significant differences. *= vs. respective vehicle (p < 0.05) and ‡= vs. respective monoculture (p < 0.05). Graphs represent data from three independent experiments.

The observed changes in ER transactivation and ER protein next led us to look for differences in cell fate when the MCF7-derived ducts were cultured alone or with fibroblasts, after exposure to a vehicle control or 100 nM E2 for 72 hours. E2 treatment as well as co-culture with stromal cells increased the total number of nuclei (Fig. 5A). To confirm this was not due to differences in adherence during cell seeding, the number of nuclei was quantified the day following seeding which revealed no difference between the monoculture and co-culture (Supplemental Fig. 2). This led us to suspect that fibroblasts were decreasing epithelial cell apoptosis or increasing proliferation.

Cell proliferation was characterized by staining for the cell proliferation marker, Ki67. E2 increased the percentage of Ki67+ cells 2-3 fold in both culture conditions. Despite the differences in confluency between the culture conditions, there was no difference in the percentage of Ki67+ cells in the monoculture compared to the co-culture (Fig. 5B). To evaluate cytotoxicity, a live/dead stain was performed on the ducts. E2 significantly increased cell viability in the monoculture platform, where 77% of cells were stained as live in the vehicle-treated monocultures compared to 93% in the E2-treated monocultures. Stromal cells also improved cell viability, where similar cell viability was observed in the E2-treated monocultures as the vehicle-treated co-cultures. There was no difference in viability between the vehicle-treated co-cultures and the E2-treated co-cultures (Fig. 5C).

Next, cell apoptosis was evaluated by staining for the apoptotic marker activated caspase-7 (CASP7). MCF7s have been reported to undergo BCL-2^30^ as well as BAX^31^ mediated apoptosis in E2 starved conditions, and CASP7 is downstream of BCL-2. E2 exposure decreased the percentage of CASP7+ cells approximately 2-fold in the monoculture platform. Fibroblasts decreased the percentage of CASP7+ cells over 5-fold, where 17% of CASP7+ cells were observed in the vehicle-treated monocultures compared to 3% in the vehicle-treated co-cultures. There was no significant difference in apoptosis between the vehicle-treated and E2-treated co-cultures, with 3% and 4% CASP7+ cells, respectively. (Fig. 5D).

As cells become less adherent when they die, we suspected that a portion of the dead cells fell off the lumen walls and into the culture media. Prior to drug feeding, culture media was collected from the lumen 24, 48, and 72 hours after drug exposure. In all culture conditions, the majority (85–90%) of cells that were in the culture medium were not viable (Supplemental Fig. 3A). Hoechst staining and quantification of the cells revealed that significantly more cells left the lumen in the vehicle-treated monocultures, compared to the other culture conditions. In monoculture, E2 reduced the amount of cells that sloughed off the lumen. The presence of fibroblasts in the collagen matrix led to a further reduction of cells washed from the lumen, where the E2-treated co-cultures had significantly less cells leave the lumen compared to the E2-treated monocultures (Supplemental Fig. 3B).

### Fibroblast co-culture prolongs epithelial confluence and increases E2-induced ductal hyperplasia

**Figure 6:**
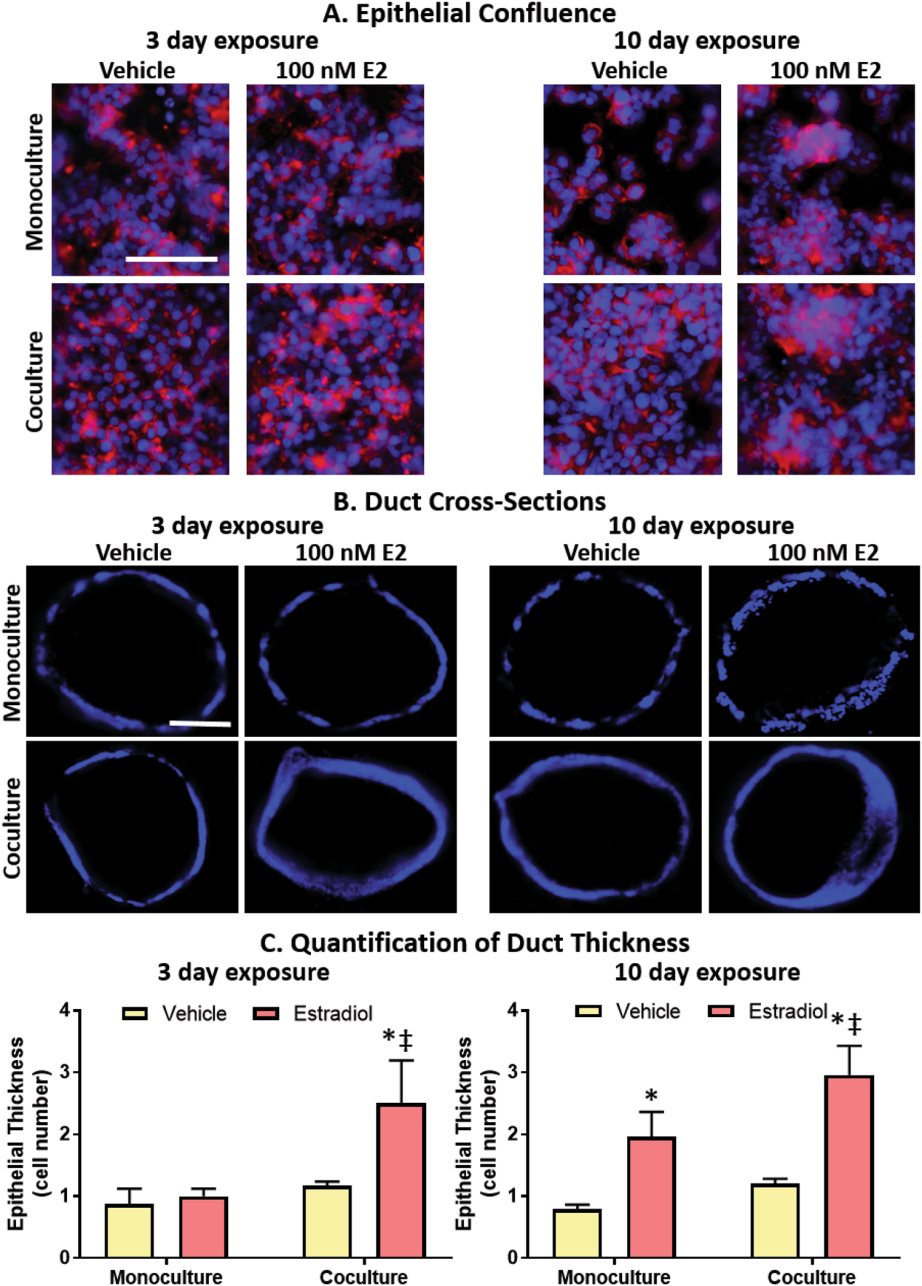
Fibroblasts prolong epithelial confluence and speed the onset as well as the severity of E2-induced hyperplasia. (**A**) Samples were stained for Hoescht (blue) and phalloidin (red) to visualize nuclei and F-actin, respectively. (**B**) To visualize hyperplasia, ducts were cross-sectioned and stained with Hoescht (blue). Scale bars represent 100 μm. (**C**) Quantification of hyperplasia. Each condition represents the cross-sections of three ducts. A two-way ANOVA was run to evaluate interactions between conditions, followed by a Tukey’s multiple comparisons test to identify significant differences. *= vs. respective vehicle (p < 0.05) and ‡= vs. respective monoculture (p < 0.05). Data is representative of three independent experiments.

As we saw a difference in apoptosis and cell number, we next tested if these cellular-induced changes were great enough to affect tissue morphology. MCF7-derived ducts grown in monoculture were compared to MCF7-derived ducts grown in co-culture, after a 3-day and 10-day exposure to a vehicle control or to 100 nM of E2. At the end of the culture period, ducts were fixed and stained for F-actin and nuclei then the bottom plane of the ducts were imaged. Despite both culture platforms exhibiting a confluent monolayer the day following seeding (Supplemental Fig. 4), MCF7-derived ducts grown in monoculture in E2-starved conditions had a visible loss of confluency after 3 days in culture, which worsened by day 10. In contrast, the monolayer was maintained in both the vehicle and E2-treated co-cultures. In both culture conditions that were exposed to estrogen, there were areas with cells piled up on top of one another, suggestive of ductal hyperplasia. (Fig. 6A).

**Figure 7:**
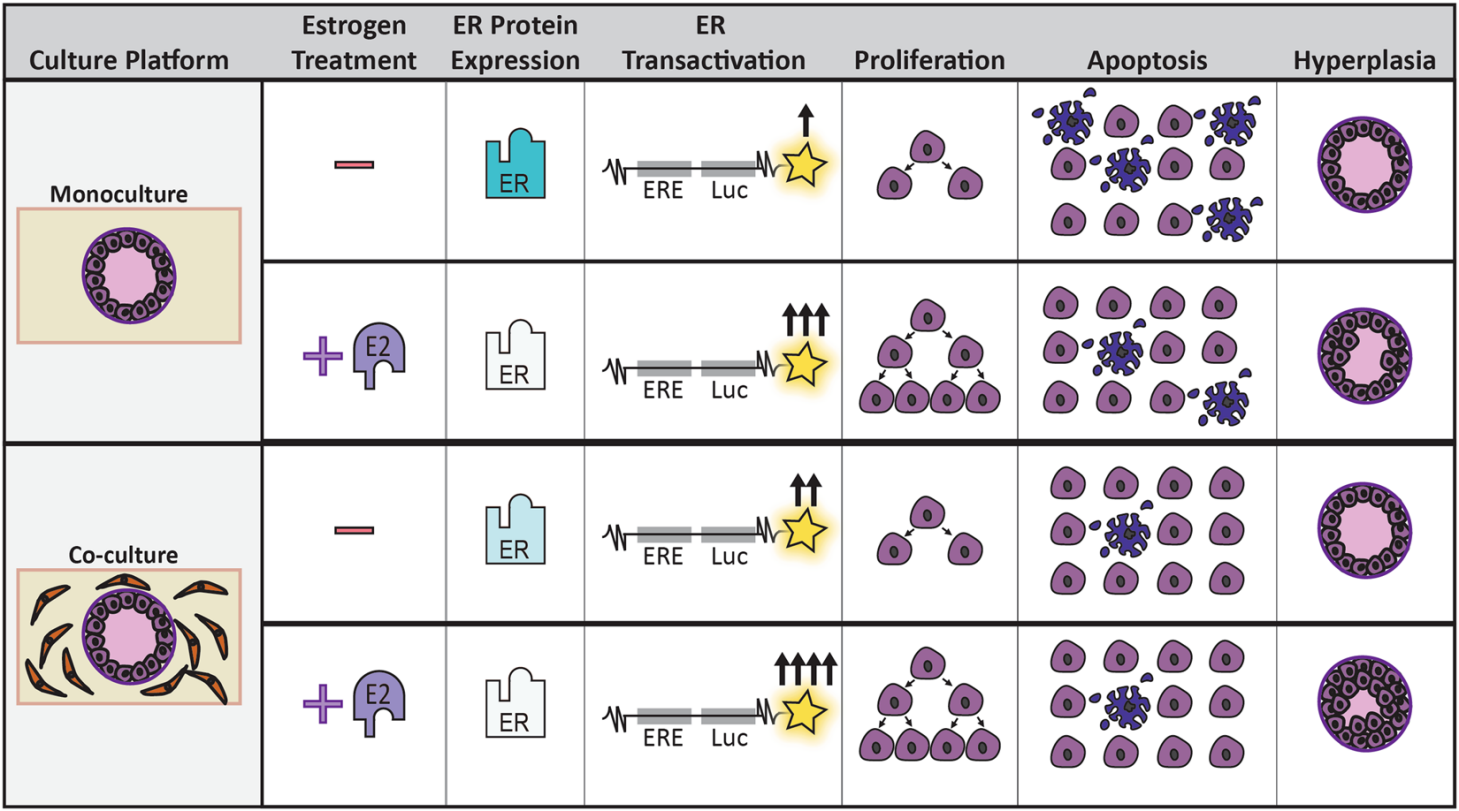
Graphical summary of major findings in the paper.

To investigate the presence of E2-induced ductal hyperplasia, MCF7-derived ducts were stained with Hoescht, embedded in agarose, cross-sectioned, and imaged. For each lumen, the number of epithelial layers lining the duct was counted at eight evenly spaced points then averaged (Fig. 6B,C). After 3 days of exposure, the ducts of the vehicle-treated monocultures, E2-treated monocultures and vehicle-treated co-cultures were lined with a single layer of cells. In contrast, the E2-treated co-culture ducts averaged approximately 2.5 cell layers. After 10 days of exposure, both the E2-treated monoculture and co-culture ducts had multiple cell layers with around 2 cell layers and 3 cell layers, respectively. A graphical summary of major results is shown in Fig. 7.

## Discussion

Despite the accepted role of ER in breast cancer progression, little is known about how different components of the tissue microenvironment modulate ER signaling or response to ER ligands. We developed an organotypic breast model based on the ER-driven breast cancer adverse outcome pathway, which helped frame which readouts (e.g. ER transactivation, apoptosis, proliferation and hyperplasia) and cellular components (e.g. fibroblasts) were most critical when modeling ER + breast cancer progression, a concept we have published previously^10^. While previous studies have introduced models that contain components of the *in vivo* mammary microenvironment^12,15,16,32–35^, we are not aware of a breast cancer model that incorporates structure, matrix proteins, and stromal cells into a single system, that is also capable of evaluating molecular, cellular and tissue level estrogenic signaling responses. Supplemental Table 2 compares characteristics of the MCF7-derived duct model introduced in this paper to organotypic mammary models described in previous publications.

Previous publications have found that MCF7s plated on plastic lack polarity, but can become polarized when cultured in a tissue relevant environment^12^. For example, one study found that MCF7s embedded in a collagen matrix were not polarized, however, the introduction of a reconstituted basement membrane (e.g. Matrigel) and stromal cells induced polarity^15^. In our platform, MCF7-derived ducts grown in normal growth media exhibited apical-basal polarity, suggesting that the ductal structure is important for cell polarization, which has been previously reported^16^. The specific mechanisms that dictate how the culture environment induces polarization have been widely discussed, and while some pathways have been identified, these interactions are not completely understood^36,37^.

The ER status of mammary fibroblasts varies between species, as well as within an individual’s mammary tissue. For example, in the adult human mammary gland, ERα is expressed in the epithelium but not the stroma^20^. In contrast, ERα is expressed in both the epithelium and stroma of the mouse mammary gland^21^. These species differences become critical when evaluating ER-driven responses, as ERα ligands could target both the stroma and the epithelium in the mouse, in contrast to just the epithelium in the human. HMFs grown in our system did not stain positively for ERα protein and did not show induction of ERα-driven genes after E2 treatment, suggesting that the fibroblasts used in our system do not express ERα. The HMFs also did not show induction of ERβ-driven genes after E2 treatment, which was evaluated because a portion of fibroblasts in the human breast expresses ERβ^38^. Overall, these results suggest that the differences in estrogenic response observed in co-culture were not due to E2 stimulating ER in the HMFs.

Previous studies using conventional 2D platforms have found that stromal cells such as cancer-associated mammary fibroblasts or bone marrow stromal cells regulate ER protein expression in mammary epithelial cells^22,23^. In this study, we found that fibroblasts also regulate ER protein in MCF7s grown as ductal structures, as demonstrated by reduced ER protein in the vehicle-treated co-cultures compared to the vehicle-treated monocultures. As ER protein is downregulated upon ER activation^28^, we suspect that the increased ER transactivation observed in the vehicle-treated co-cultures was responsible for the reduction in ER protein. Surprisingly, ER protein was similar in both the estrogen-treated monoculture and co-cultures, which is notable because ER transactivation was significantly higher in the estrogen-treated co-cultures compared to the estrogen-treated monocultures. This suggests that ER signaling dynamics are distinctly different in MCF7s cultured with fibroblasts, compared to MCF7s cultured alone. These differences could become critical when evaluating breast cancer therapeutics and risk factors because drug resistance/sensitivity could change in the presence of stromal cells.

Our finding that MCF7 cell density was increased in the presence of fibroblasts is consistent with previous findings^23^, and led us to hypothesize that fibroblasts were influencing proliferation and/or apoptosis. Upon characterization of the cell proliferation marker Ki67, we found the percentage of Ki67+ cells in the MCF7-derived ducts (~2–4% Ki67+ cells) was comparable to the percentage of Ki67+ cells in normal human breast tissue (~3% Ki67+ cells)^39^. These percentages are dramatically reduced compared to cells cultured in 2D, where one group found that 90% of MCF7s plated on plastic are Ki67+ ^40^. Similar to previous reports^41^, E2 increased MCF7 proliferation, as indicated by a 2–3 fold increase in Ki67 expression. Despite finding an increased cell density in our co-culture platform, we did not observe a difference in Ki67+expression in our monoculture vs. co-culture, leading us to suspect that the differences in cell density were independent of proliferation.

Previous publications have found that some ER + breast cancer cells rely on estrogen for survival, and undergo apoptosis when grown in estrogen-starved conditions^31,42^. Therefore, it was not surprising that a portion of the MCF7s treated with a vehicle control, and consequently estrogen-starved, were dead or undergoing apoptosis. Also in agreement with previous publications^30^, E2 treatment improved cell viability and reduced apoptosis in our monoculture platform. When fibroblasts were included in the matrix, there were low levels of apoptosis and high cell viability even in the absence of estrogen. We did not observe significant E2-induced differences in cytotoxicity or apoptosis in co-culture; however, we suspect that E2 had no effect on cell viability in the co-cultures because the paracrine signals from the fibroblasts increased viability to such high levels (approaching 100%) that viability could not be further increased by estrogen treatment. This suspicion is supported by the concomitant low levels of apoptosis (~3% cells) in both the vehicle and estrogen-treated co-cultures. Future studies are needed to determine if the estrogen- and paracrine-induced reduction of apoptosis occur through shared or distinct mechanisms, however, the increased ER activity in conjunction with the reduced apoptosis in our co-cultures suggests an ER-dependent mechanism. Upon examination of MCF7s that detached from the lumen due to cell death, we found that significantly less cells left the lumen in the E2-treated co-cultures compared to the E2-treated monocultures. We suspect that this reduction in cell loss, along with the maintenance of E2-induced proliferation, could explain the increased cell density observed in the co-cultures.

We characterized ductal hyperplasia, which refers to the abnormal growth of epithelial cells into the center of a breast duct, because ductal hyperplasia is commonly used in a clinical setting as it is a defining feature of premalignant breast lesions^43^. 2D *in vitro* models are unable to evaluate hyperplasia due to the absence of tissue structure. In the monoculture platform, hyperplasia was observed after 10 days of E2 exposure, which agrees with other *in vitro* organotypic studies that reported ductal hyperplasia in the presence of E2^12,34^. When stromal cells were incorporated into the matrix, E2-induced hyperplasia was detected sooner and was more prominent than in our monoculture model, likely due to reduced apoptosis and consequently the increased cell density observed in the co-culture platform (refer to Fig. 7 for graphical summary).

Our finding that HMFs modulate both ER protein and apoptosis in MCF7 cells may shed light into the perplexing role of ER-regulated apoptosis. Treatment with anti-ER therapies typically induces apoptosis in ER + breast tumors, and exposure to ER agonists reduces apoptosis. However, breast tumors can become resistant to anti-ER therapies, and in these tissues, exposure to ER agonists can increase apoptosis^44,45^. It is not clear what causes a tumor to respond differently to ER ligands, however, some have speculated that the interactions between the tumor cell and the surrounding stroma play a role^46,47^. Therefore, future studies that evaluate how primary fibroblasts or other mammary fibroblast cell lines affect ER-driven responses in the MCF7-derived duct system may provide insight into the role of the stroma in mediating therapy resistance and its relation to ER-regulated apoptosis. Of course, these future studies must consider the shortcomings associated with the MCF7-derived duct model. For example, the model is less throughput than traditional *in vitro* models, due to the incorporation of the biomimetic ductal structure, matrix proteins and additional cell types. Additionally, the overall complexity of the model introduces additional variables that must be considered when investigating biological or chemical mechanisms.

In summary, MCF7s cultured in a mammary duct structure exhibited apical-basal polarity, cellular proliferation levels similar to the ducts of a normal breast *in vivo*, and growth into the center of the lumen resembling atypical ductal hyperplasia. We confirmed that the MCF7-derived duct model responds to estrogens similarly to what has been previously shown both *in vitro* and *in vivo*, where E2 exposure induced ductal hyperplasia, increased proliferation, decreased apoptosis, increased ER transactivation and reduced ER protein. Incorporation of fibroblasts into the surrounding matrix sped the onset and increased the severity of estradiol-induced hyperplasia. We found the increased hyperplasia was due to MCF7s exhibiting lower levels of apoptosis coupled with similar levels of estradiol-induced proliferation in co-culture, which led to an increased cell density. Furthermore, the reduction in apoptosis may have occurred through an ER-dependent mechanism, as we observed a reduction in ER protein and an increase in ER transactivation in the co-culture model. Overall, this study introduces a new platform that can be used to evaluate the role of the mammary microenvironment in ER + breast cancer progression, and provides insight into the importance of fibroblasts when evaluating environmental chemicals or candidate therapeutics.

## Materials and Methods

### Cell culture

We used a variant of the immortalized ER + human mammary epithelial cell line MCF7, MVLN (referred to as MCF7s) that has previously been stably transfected with an ERE-luciferase construct^29^. Immortalized human mammary fibroblasts (referred to as HMFs) derived from the stromal vascular fraction of a reduction mammoplasty^48^ were gifted to us from Dr. Lisa Arndt’s lab (University of Wisconsin, Madison). MCF7s and HMFs were grown in high glucose DMEM (4.5 mg/ml, Gibco, Gaithersburg, MD, USA; #11965092) supplemented with 10% fetal bovine serum (FBS, VWR #97068-085) and 1% penicillin/streptomycin (Thermo Fisher, Waltham, MA, USA; #15140-122) and maintained in a 5% CO_2_ 37 °C incubator.

### Device fabrication

Fabrication of the LumeNEXT microfluidic devices has been described previously^49^. Briefly, standard photolithography methods were employed to create the SU-8 masters that were used as molds for the polydimethylsiloxane (PDMS) (Dow Corning, Auburn, MI, USA; Sylgard 184 Silicone Elastomer Kit #3097358-1004) device. After the two layers of the devices were bonded, 340-μm diameter PDMS rods were placed into the device chamber. The device was oxygen-plasma-treated and bonded to a 48 × 65 mm Gold Seal cover slip (Thermo Fisher, 48 × 65-1-002) that was taped to an omnitray (Thermo Fisher #242811). Once the device was bonded, the middle chamber of the device was closed to the air and facing the glass, while the device ports were open (fabrication shown in Fig. 1A). A single 86 × 128 mm omnitray can hold up to 36 lumen devices (example of a 24 lumen array shown in Fig. 1D). Devices were UV sterilized for 15–20 minutes then transferred to a biosafety hood.

### Organotypic culture preparation

To minimize evaporation, sterile Kimwipes soaked in PBS were placed along the corners of the Omnitray that held the devices. To aide collagen attachment to the device, a 2% poly(ethyleneimine) (PEI) (Sigma-Aldirch, St. Louis, MO, USA; #03880) solution diluted in deionized (DI) water was loaded into the side ports and incubated at room temperature for 10 minutes. The PEI solution was removed from the channel and a 0.4% glutaraldehyde (GA) (Sigma #G6257) solution diluted in DI water was loaded into the side ports and incubated at room temperature for 30 minutes. While the GA incubated, a collagen solution was prepared on ice. High-density rat-tail collagen type 1 (Corning, Corning, New York, USA; #354249, will refer to as collagen for the remainder of the paper) was diluted with 10 × PBS and neutralized with 0.5 M NaOH to a final concentration of 6 mg/ml collagen, a final concentration of 1 × PBS, and a pH of 7.2. After the GA incubation, the devices were washed three times with DI water. Directly prior to collagen loading, the 6 mg/ml collagen solution was diluted 3 to 1 in media (for the monoculture) or in a 5,000 cell/μl fibroblast cell solution (for the co-culture). The final 4.5 mg/ml collagen solution was loaded into the side ports and incubated at room temperature until the collagen turned white, then transferred to the incubator for one hour. A small drop of media was placed into the input port and the PDMS rod was pulled out through the output port, leaving a hollow lumen structure that connected the input and output port. 1.5 μl of a MCF7 solution (~50,000 cells/μl) was pipetted into the input port and into the lumen. Devices were placed in the incubator and flipped from bottom to top every 20 minutes for 100 minutes. The output port was aspirated and filled with media then devices were cultured upside down overnight. The next morning, media was flowed through the input port then aspirated out of the output port to clear the lumen of dead or unattached cells. The media was changed every day. Device design and culture conditions are illustrated in Fig. 1.

### Hormone treatments

Prior to all estrogen experiments, cells were grown in estrogen-deprived media containing phenol red-free DMEM (Thermo Fisher #31053-028) supplemented with 10% charcoal dextran stripped FBS, 1% penicillin/streptomycin and 1% L-Glutamine for 48 hours. Where hormone treatment is indicated, a vehicle (0.1% ethanol) control or hormone treatment (estradiol (Sigma-Aldrich, #E2758) or diethylstilbestrol (Sigma-Aldrich #D4628)) at the indicated concentrations were added directly to the input port of the lumens and replenished daily.

### Immunofluorescence staining

Cells were fixed with 4% paraformaldehyde (PFA) (Alfa Aesar, Tewksbury, MA, USA; #43368) then washed with PBS three times. To visualize filamentous actin (F-actin), cells were stained with Texas red phalloidin (1:200, Thermo Fisher #T7471). For all staining experiments, cells were stained with Hoechst (1:500, Thermo Fisher #H3570) to visualize nuclei. For intracellular stains, cells were permeabilized with 0.2% Triton for 20 minutes then blocked with 3% BSA overnight. To evaluate ERα expression in 2D, cells were stained for ERα (1:200, anti-mouse, Thermo Fisher #MA5-13304) overnight, washed with 0.1% TWEEN three times, and incubated with Alexa flour 488 (1:200, anti-mouse; Abcam, Cambridge, MA, USA) for 2 hours. Cultures were washed 3 times with 0.1% TWEEN 20 diluted in PBS then imaged. The following antibody stains were performed in our organotypic cultures and have different dilutions/staining times than cells plated in 2D. To evaluate apical-basal polarity, cells were stained for basal marker laminin-5 (1:50, anti-mouse, Abcam #ab78286) and apical marker GM130 (1:50, anti-rabbit, Abcam #ab52649). To evaluate proliferation, cells were stained for proliferation marker Ki67 (1:50, anti-rabbit, Thermo Fisher #RM9106-S). To evaluate ER expression, we used the same ER antibody as in our 2D ER staining protocol. After cells were incubated with primary antibodies for 48 hours, cells were washed 5 times over a 24-hour period with 0.1% TWEEN 20 diluted in PBS. Next, cells were incubated with Alexa Flour 488 (1:50, anti-mouse, Abcam #ab150113) and/or Alexa Flour 647 (1:50, anti-rabbit, Thermo Fisher #a-21244) for 2 days. The samples were then washed 5 times over a 24-hour period then imaged.

### RT-qPCR

Prior to mRNA isolation, MCF7s and HMFs were grown on a 96 well plate and exposed to a vehicle control or 10 nM E2 for 24 hours. mRNA isolation was performed with Dynabeads mRNA direct purification kit (Thermo Fisher #61011) and reverse transcription was done with the high capacity RNA to cDNA kit (Thermo Fisher #4387406). RT-qPCR was conducted using Taqman Probes (Thermo Fisher). The ΔΔCT method was used to evaluate relative gene expression, where expression of ER-driven genes ESR1 (Hs00174860_m1), TFF1 (Hs00907239_m1), PGR (hs01556702_m1), and JAG1 (Hs01070032_m1) were normalized to housekeeping genes GAPDH (Hs99999905_m1) and HPRT (11501003267_m1). A student’s t test was used to evaluate significant differences between vehicle and E2 treated cultures (defined as p > 0.05). Graphs displayed are representative of data normalized to the GAPDH housekeeping gene, although the same findings were observed when the data was normalized to HPRT.

### Live/dead assay

MCF7-derived ducts were washed with serum-free phenol red-free DMEM then replaced with a solution containing calcein AM (2:500, Thermo Fisher #c3100mp), ethidium homodimer-1 (2:500, Thermo Fisher #e1169), and Hoescht (1:500) diluted in serum-free phenol red-free DMEM for 30 minutes at 37 °C.

### Apoptosis assay

CellEvent Caspase 3/7 Green Detection Reagent (1:1000, Thermo Fisher #C10423) and Hoescht (1:500) were diluted in estrogen-free media and incubated with the cells at 37 °C for 30 minutes. MCF7s do not express caspase-3^50^ so apoptosis identified by this assay is indicative of caspase-7 activity only.

### Evaluation of cell loss

Starting 24 hours after the first dosing period, media was collected from lumens daily prior to cell feeding and continued until the end of the 72-hour dose period. Media was pipetted from the large port then transferred to a 96 well plate containing the live/dead staining solution described above. Number and viability of collected cells was quantified at each day of collection. For each technical replicate, the number of collected cells was summed to estimate the total cell number that left the lumen over the course of the 3 days. Likewise, for each technical replicate, the viability of collected cells was averaged to estimate the viability of cells that left the lumen over the course of the 3 days.

### Lumen cross sectioning and quantification

After the lumens were fixed and stained, the lumen was aspirated and filled with 3% low melting point agarose (IBI Scientific, Peosta, Iowa, USA; #IB70056) diluted in PBS to prevent the lumen from collapsing during cross-sectioning. Once the agarose solidified, tweezers were used to remove the top layer of the PDMS device then the sample was completely embedded in agarose. The sample was glued to a mounting block, with the lumen faced orthogonal to the mounting block, then sliced with a VT-300 Compresstome (Precisionary Instruments, Greenville, North Carolina, USA). The resulting 100-μm thick cross-sections were immediately imaged. For each cross-section, hyperplasia was quantified by counting the number of nuclei lining the duct (referred to as epithelial thickness) at eight evenly spaced points then averaging the epithelial thickness.

### Image acquisition

All images were taken with fluorescent microscope Nikon Eclipse Ti. Unless otherwise indicated, images were taken of the bottom plane of the lumen.

### Fluorescent image quantification

The automated image-processing program, JeXperiment^51^ was used for image quantification. First, we conducted a rolling ball background subtraction. Region of interests (ROIs) were drawn over the lumens in one Z-plane to exclude fibroblasts and minimize noise. The ROI dimension and background subtraction was kept constant throughout a dataset.

To count total nuclei, CASP7+ nuclei, as well as Ki67+ nuclei, we counted all maxima within the ROI defined by a set threshold. To evaluate the percentage of Ki67+ cells and the percentage of CASP7+ cells, the number of Ki67+ or CASP7+ nuclei was divided by the total number of nuclei for each sample. The percentage of viable cells was determined by dividing the number of viable cells by the total number of nuclei.

ER protein was quantified as described previously^23^. Briefly, single cell analysis was performed on samples stained for nuclei and ERα protein, and was used to quantify ERα protein levels per individual cell. First, masks outlining the nuclear stain Hoescht were generated. The nuclear masks were overlayed on top of the ERα images and the ER staining intensity within each nuclear mask was quantified. We presented the data in two ways. For each culture condition, we graphed the means of ER expression to gain an understanding of how the intensity of ERα expression changes with the addition of estrogen as well as fibroblasts. Second, we created a density plot in the statistical program R to describe the distribution of ERα protein expression within each population.

### Luciferase transactivation assay

Prior to conducting the luciferase assay, samples were incubated at room temperature for 10 minutes. 6 ul of 1 mM beetle luciferin (Promega, Fitchburg, Wisconsin, USA; #E1601) solution diluted in estrogen-deprived media was added into the input port and luminescence was quantified with a Biorad Chemidoc imager. Luminescence is indicative of ER transactivation, as characterized previously and described above^29^.

### Statistics

Graphpad Prism was used for statistical analysis. Any lumens that were not viable the day following cell seeding were excluded from analysis. A two-way ANOVA followed by a multiple comparisons test was used to determine significance (as defined by p < 0.05) for experiments evaluating how estrogen exposure in addition to stromal cells influenced an endpoint. ANOVA interaction tables are summarized in Supplemental Table 1. We used nonlinear regression (equation: Y = Bottom + (Top-Bottom)/(1 + 10^((LogEC50-X)*HillSlope))) to determine EC50s for dose response curves. Data shown is representative of multiple experiments, as indicated in the graphs. Error bars represent standard deviation in all graphs except for the dose response graphs, where standard error was used.

## Electronic supplementary material

Supplementary Information
