# Supplementary material for: Mammary fibroblasts reduce apoptosis and speed estrogen-induced hyperplasia in an organotypic MCF7-derived duct model
Source: Sci Rep. 2018 May 8;8:7139. doi: 10.1038/s41598-018-25461-1 (PMC5940820; doi:10.1038/s41598-018-25461-1)
Supplement: Supplementary file 1 — Supplementary Information [file 41598_2018_25461_MOESM1_ESM.pdf]

Supplemental Files

Mammary fibroblasts reduce apoptosis and speed estrogen-induced hyperplasia in an organotypic MCF7-derived duct model

Molly M. Morgan[a], Megan K. Livingston[a,b], Jay W. Warrick[a], Eli M. Stanek[a], Elaine T. Alarid[c], David J. Beebe[a,*], Brian P. Johnson[a,*]

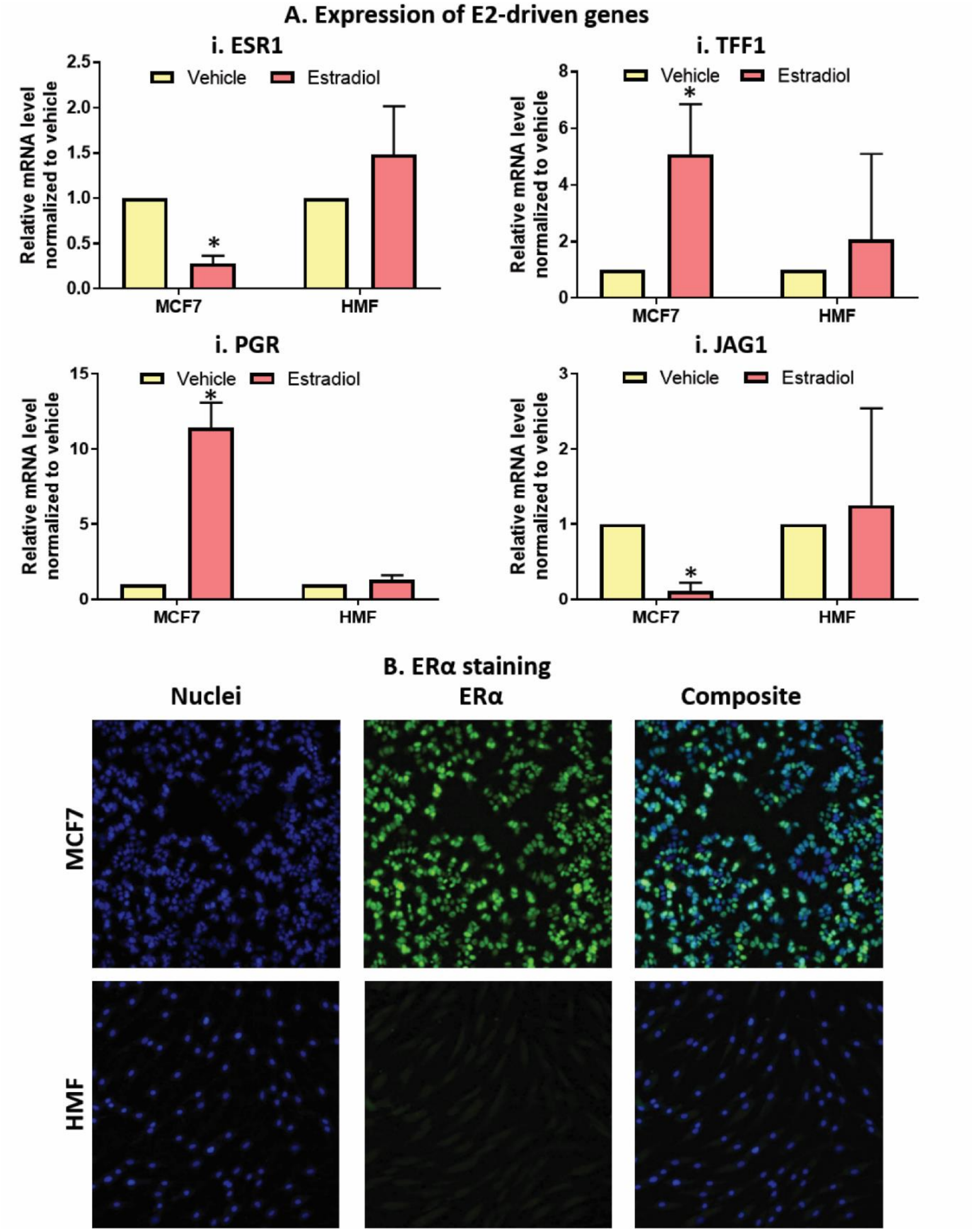

Supplemental Figure 1. **ERα- and ERβ- driven genes are not induced in HMFs after E2 exposure, and HMFs do not stain positively for ERα protein.** **(A)** After MCF7 cells and HMF cells were exposed to E2 for 24 hours, qRT-PCR was used to evaluate expression of ERα-driven genes *ESR1*, *TFF1*, and *PGR* and ERβ-driven gene *JAG1*. Each sample was normalized to the expression of the housekeeping gene *GAPDH*. A student's t test was used to evaluate significance. * = vs. respective vehicle ($p < 0.05$). **(B)** MCF7 cells and HMF-621-tert cells were stained for ERα and nuclei.

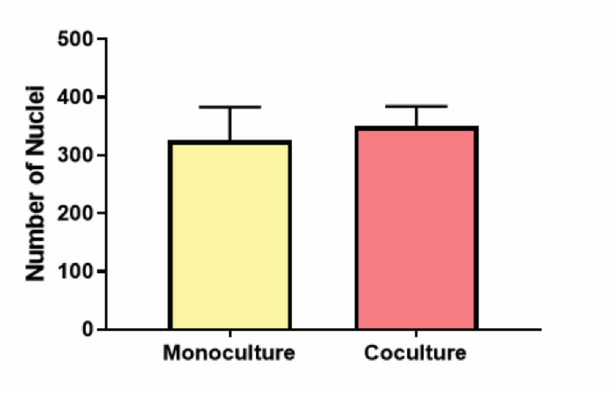

Supplemental Figure 2. **Cell number is similar in monoculture and co-culture the day following seeding.** The day following cell seeding, samples were fixed and stained for nuclei to quantify cell number.

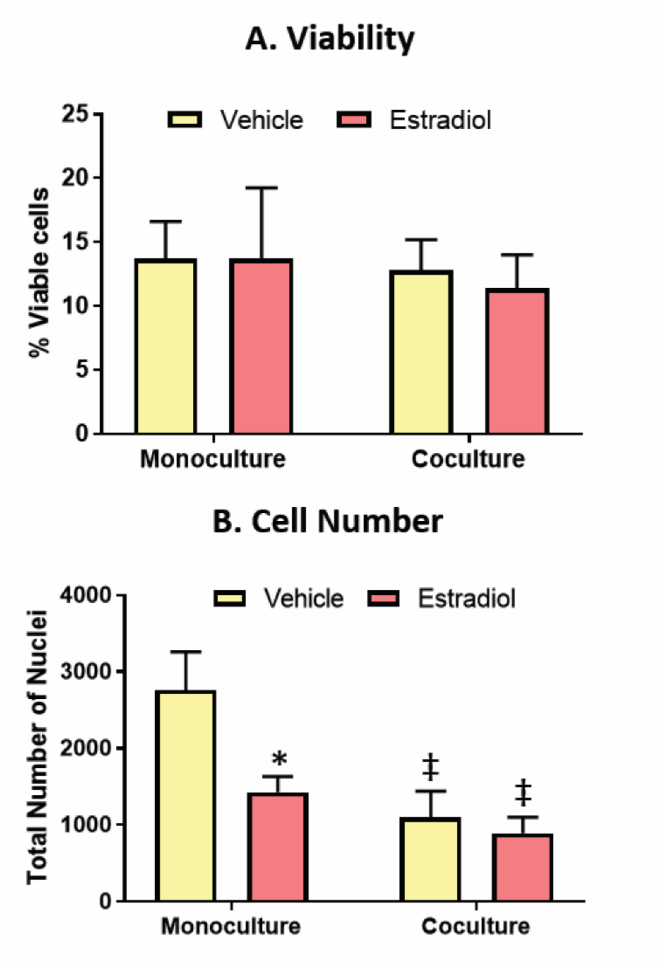

Supplemental Figure 3. **Estrogen as well as fibroblasts reduced the number of dead cells leaving the lumen.** Culture media was collected from lumens after 24, 48, and 72 hours of estrogen exposure. **(A)** Cell viability was measured by determining the amount of live (calcein positive) cells relative to the total cell population. The viability shown is the average viability of the 24, 48, and 72-hour dose periods. **(B)** Cell number was evaluated by staining for nuclear marker Hoescht and quantifying the total number of nuclei. Cell number shown is the sum of cells lost during the 24, 48, and 72-hour time points.

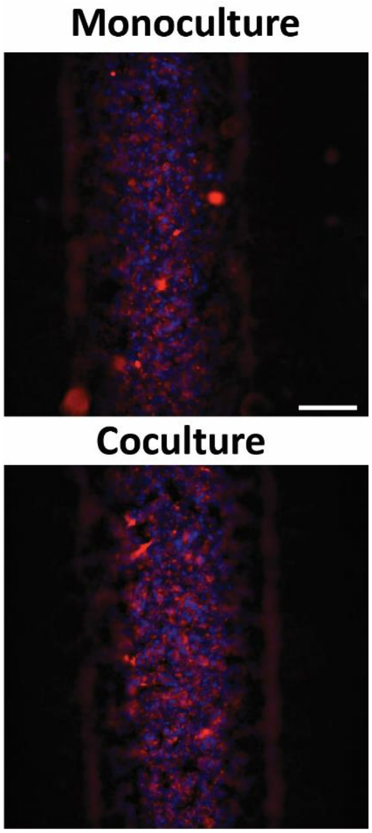

Supplemental Figure 4. **Lumen confluence of monoculture and co-culture appeared similar the day following seeding.** The day following seeding, samples were fixed and stained with phalloidin (red) and Hoescht (blue) to visualize F-Actin and nuclei, respectively. Scale bar represents 100 μm.

Endpoint	Culture Condition	Estrogen Treatment	Interaction
**ER Transactivation**	<0.0001	<0.0001	0.0005
**ER Protein**	0.0083	<0.0001	0.0418
**Number of Nuclei**	<0.0001	<0.0001	0.6148
**Proliferation**	0.2597	<0.0001	0.3561
**Cytotoxicity**	0.0005	0.0006	0.0226
**Apoptosis**	0.0006	0.0226	0.0046
**Number of Cells Lost**	<0.0001	<0.0001	0.0011
**Viability of Cells Lost**	0.338	0.6653	0.6653
**Hyperplasia (3 Day Exposure)**	0.0034	0.01	0.024
**Hyperplasia (10 Day Exposure)**	0.0045	<0.0001	0.147

Supplemental Table 1. **Summary of two-way ANOVA results.**

Publication	Estrogen Responsive	Presence of Hyperplasia	Polarized Epithelial Cells	Stromal Component	Method Used to Acquire Tissue Structure	ECM
**Morgan, 2018**	Yes, E2 affected ER protein, ER transactivation, cell number, proliferation, cytotoxicity, apoptosis, and hyperplasia	Yes	Yes	Fibroblasts	Seeded into engineered lumen; formed lumen structure	Yes; Collagen
**Bischel, 2015**	Not evaluated	Not evaluated	Yes	Fibroblasts	Seeded into engineered lumen; formed lumen structure	Yes; Mixture of collagen and Matrigel
**Carter, 2017**	Not evaluated	Yes	Yes	Myoepithelial cells	Self-assembled into bilayered luminal structures	Yes; Collagen or Matrigel
**Debnath, 2003**	Not evaluated	Not evaluated	Yes	No	Self assembled into luminal structures	Yes; Matrigel
**Krause, 2010**	Not evaluated	Yes	Not polarized in collagen only. Polarized with the addition of reconstituted basement membrane or fibroblasts	Fibroblasts	Self-assembled into spheroids. Addition of reconstituted basement membrane or fibroblasts resulted in lumen formation	Yes; Collagen or Matrigel
**Marchese, 2012**	Yes, E2 affected apoptosis, cell number, hyperplasia	Yes	Yes	No	Self assembled into luminal structures	Yes; Matrigel
**Vantangoli, 2015; Vantangoli, 2017**	Yes, E2 affected gene expression and hyperplasia	Yes	Hemi-polarized	No	Self-assembled into microtissues that contained a luminal space	No; Agarose scaffold

Supplemental Table 2. **Comparison of MCF7-derived duct model to previously published organotypic breast models.**
